# Development of a multiplex quantitative PCR assay for simultaneous detection of *Treponema phagedenis, Treponema pedis, Treponema medium*, and *‘Treponema vincentii’* and evaluation on bovine digital dermatitis biopsies

**DOI:** 10.1007/s11259-023-10147-5

**Published:** 2023-06-01

**Authors:** Sara Frosth, Hanna K. Eriksson, Anna Rosander

**Affiliations:** 1 Department of Biomedical Sciences and Veterinary Public Health, Faculty of Veterinary Medicine and Animal Science, Swedish University of Agricultural Sciences (SLU), P.O. Box 7036, Uppsala, 750 07 Sweden; 2 Department of Animal Nutrition and Management, Faculty of Veterinary Medicine and Animal Science, Swedish University of Agricultural Sciences (SLU), P.O. Box 7024, Uppsala, 750 07 Sweden

**Keywords:** Bovine digital dermatitis, Contagious ovine digital dermatitis, Ovine footrot, Lameness, Cattle, Sheep, Real-time PCR

## Abstract

Bovine digital dermatitis (BDD) is a contagious foot disease with worldwide occurrence in dairy cattle. The disease causes lameness and reduced animal welfare as well as economic losses for the farmer. The aetiology is not fully established but associations have been made with *Treponema* spp. Today, BDD diagnosis is mainly based on visual inspection of cattle feet, therefore this study aimed to develop a multiplex quantitative PCR (qPCR) assay targeting *Treponema phagedenis*, *Treponema pedis, Treponema medium*, and *‘Treponema vincentii’* to aid in diagnosis. The assay was tested for specificity on 53 bacterial strains and *in silico* on 168 *Treponema* spp. genomes, representative of at least 24 species. In addition, 37 BDD biopsies were analysed and the results compared to another qPCR assay published during the study period, which we modified by combining into a multiplex qPCR. The qPCR developed herein had a detection limit of 10 copies of each target species per PCR reaction. Both qPCR assays showed 100% specificity when tested on bacterial strains, but the qPCR developed in this study detected 3.4% more *T. phagedenis*-positive biopsies of lesion category M1-M4.1 than the modified assay. To conclude, the developed qPCR assay detecting *T. phagedenis*, *T. pedis, T. medium*, and *‘T. vincentii’* has high analytical sensitivity and specificity and provides a useful complementary tool for diagnosis and epidemiological studies of BDD. The assay could possibly also be used for contagious ovine digital dermatitis (CODD) as similar bacteriological profiles have been suggested for BDD and CODD, especially regarding certain *Treponema* spp.

**Supplementary Information:**

The online version contains supplementary material available at 10.1007/s11259-023-10147-5.

## Introduction

Bovine digital dermatitis (BDD) and contagious ovine digital dermatitis (CODD) are infectious foot diseases causing lameness in cattle and sheep, respectively. The aetiology of these diseases is not fully understood, but *Treponema* spp. and in particular, *Treponema phagedenis, Treponema medium*, and *Treponema pedis* have been found to be associated with both diseases (Evans et al. 2009; Sayers et al. 2009; (Sullivan et al. 2015a, b). Recently, it has been suggested that CODD arises from footrot lesions and that *Dichelobacter nodosus* and *Fusobacterium necrophorum* play a part in the aetiology in addition to the three treponemes (Staton et al. 2021).

In Sweden, the occurrence of BDD has increased since it was first described in 2005 (Hillström and Bergsten 2005), and is now relatively common in dairy cattle – BDD was found on 55% of Swedish cattle farms in 2020, and BDD lesions were observed at 4.9% of individual trimmings (F. Åkerström, Växa Sverige, personal communication April 20, 2022). Besides being an animal welfare problem, BDD contributes to substantial economic losses due to e.g. reduced milk production, weight loss, and early culling of otherwise productive animals (Bruijnis et al. 2010; Cha et al. 2010). CODD was not discovered in the country until 2019 and has so far only been detected in two Swedish sheep flocks (Bernhard et al. 2021). The gold standard for BDD and CODD diagnosis is visual inspection of lifted hooves (Afonso et al. 2021). Effective laboratory diagnostics can aid in clinical diagnosis and be helpful in epidemiological studies in general. However, it is an advantage if the methods are quick and easy to perform. A conventional PCR has already been described for detection of three BDD-associated phylogroups (*T. phagedenis*-like, *T. medium/T. vincentii*-like, and *T. denticola/T. putidum*-like) (Evans et al. 2009), but it is not run in multiplex and requires subsequent agarose gel electrophoresis. Quantitative PCR (qPCR) methods, which are faster and more convenient for analysis of larger numbers of samples, have also been developed for the simultaneous detection of three and four *Treponema* species, respectively (Anklam et al. 2017; Beninger et al. 2018). However, in our hands, the triplex assay (Anklam et al. 2017) showed signs of cross-reactivity and the fourplex (Beninger et al. 2018) failed to amplify the type strains of *T. medium* (ATCC 700293^T^) and *T. pedis* (DSM 18691^T^). More recently, qPCR assays targeting the recombinase A (*recA*) genes of *T. phagedenis*, *T. pedis*, and *T. medium* have been developed by Staton et al. (2021), but they were all run in singleplex.

Therefore, this study aimed to develop a multiplex qPCR assay for the simultaneous and specific detection of *T. phagedenis, T. pedis, T. medium*, and *‘T. vincentii’* and to evaluate this method on BDD samples. A comparison between the triplex qPCR developed in this study and a modified version of the *recA*-targeting qPCR assays by Staton et al. (2021) was also performed.

## Materials and methods

### Multiplex quantitative PCR (qPCR) assay design

The PrimerQuest™ Tool (Integrated DNA Technologies (IDT), Inc., Coralville, IA, USA) available at https://www.idtdna.com/SciTools, accessed on 7 April 2021), was used to design the qPCR primers and TaqMan probes (Owczarzy et al. 2008). The assay specific for *T. phagedenis* targeted the *vspA* gene and was based on the following accession numbers: CP042813.1-CP042818.1, CP054692.1, CP058241.1 and KU143867.1- KU143879.1. The following accession numbers were used to design the assay targeting the TPE 0673 gene of *T. pedis*: CP004120.1, CP061839.1, NZ_AOTQ01000078.1, NZ_AOTM01000086.1, NZ_AOTN01000018.1 and NZ_AOTR01000048.1. For detection of *T. medium* and *‘T. vincentii’* the same primers/probe targeting the 16S rRNA gene were used due to high similarity between the species. Therefore, from now on and throughout the manuscript we use the term *T. medium/‘T. vincentii’* for this assay since they are detected together. The *T. medium/‘T. vincentii’* assay was based on accession numbers: CP031393.1, EF061249.1-EF061252.1, FJ204241.1, FJ204242.1, GU420876.1, GU420877.1, KP063153.1, KP750180.1, KP859544.1-KP859546.1, KR025809.1-KR025819.1, KR025825.1, KR025853.1 and KT192148.1. The qPCR primers and TaqMan probes were checked for possible secondary structures and dimerizations by the OligoAnalyzer™ Tool (IDT), also available at https://www.idtdna.com/SciTools (Owczarzy et al. 2008). The specificity of the assays was checked by BLAST® https://blast.ncbi.nlm.nih.gov/Blast.cgi (Altschul et al. 1990). The designed and evaluated qPCR primers and TaqMan probes with sequences, fluorophores, and quenchers in Table 1 were ordered from IDT, except the TaqMan probe for *T. medium/‘T. vincentii’*, which was ordered from Thermo Fisher Scientific Inc. (Waltham, MA, USA). The amplicons for the three different targets were between 104–147 base pairs (bp) in length (Table 1).

**Table 1:** Primers and TaqMan probes designed for quantitative PCR analysis

Specificity	Name	Sequence 5’-3’	Amplicon (bp)
*T. phagedenis*	*vspA*-F	AAACACTGGTGGTAAGGTTCA	111
	*vspA*-R	GTTCCGCCTAGTGGTGTATTT	
	*vspA*-P	5TEX615-TCCAGGTGAAAGCGGTAACACATCC-3IAbRQSp	
*T. pedis*	TPE 0673-F	GTACGGCTTGTATTACGATGGA	104
	TPE 0673-R	CCAGGTTTAAGCGTACTACTCC	
	TPE 0673-P	5Cy5-ACGGGAACA/TAO/AACGGAGTAACACGA-31AbRQSp	
*T. medium/ ‘T. vincentii’*	16S-F	TCTAGTAGAAGGTCTTAGAGATAAGGC	147
	16S-R	AGCTTTACCTGTTAGTAACTGGCAG	
	16S-P	6FAM-TAGCAATACCCTGCTAGAC-MGBNFQ	

### Preparation of qPCR standards

A pUC57 plasmid containing the three-amplicon sequences for *vspA*, TPE 0673, and *T. medium/‘T. vincentii’* 16S, separated by “AATAA” as spacers, was ordered from Genscript Biotech Corporation (Piscataway, NJ, USA) with *Bam*HI and *Xho*I sites added to the terminals of the target sequence to enable downstream linearization (digestion by restriction enzymes). Linearization of plasmid standards has been shown to be important to avoid overestimation of copy numbers in absolute quantification (Hou et al. 2010). Absence of *Bam*HI and *Xho*I sites in the target sequence was checked with NEBcutter V2.0 (Vincze et al. 2003). Upon arrival of the 100-µg plasmid preparation, the lyophilized plasmid was dissolved in 100 µl nuclease-free water (W4502; Sigma–Aldrich, St Louis, MO, USA) before 10 µg was cleaved with 20U *Bam*HI (New England Biolabs Inc., Ipswich, MA, USA) in 1 × NEBuffer 2 (New England Biolabs Inc.) for 2 h at 37 °C. The linearized plasmid was purified using the QIAquick PCR Purification Kit (Qiagen, Hilden, Germany) according to the manufacturer’s instructions with some modifications. The washing step was performed twice instead of once and the elution was performed with pre-warmed buffer EB (70 °C) that was allowed to incubate on the column at 70 °C for 3 min prior to centrifugation. The linearized and purified plasmid was then analysed on a 1% agarose gel together with uncleaved plasmid to verify that it had been cleaved and a distinct band of the correct size (3088 bp) was visualised using GelRed (Sigma–Aldrich) and UV light.

The DNA concentration of the linearized plasmid was determined using the Qubit dsDNA Broad Range Assay Kit (Invitrogen, Carlsbad, CA, USA) in a Qubit® 2.0 Fluorometer (Invitrogen). Plasmid copy number was calculated using the following formula: number of copies per µl = (Plasmid concentration (ng/µl) × Avogadro’s number)/(length of plasmid (bp) × average weight of a base pair (Da) × conversion factor to ng). The total length of the plasmid including the 387-bp insert used in this study was 3088 bp. The linearized plasmid was diluted to 50,000 copies/µl and then ten-fold serial dilutions were made in nuclease-free water (W4502; Sigma–Aldrich) containing 1 ng/µl Polyinosinic–polycytidylic acid potassium salt (P9582; Sigma–Aldrich) as a stabiliser down to 0.5 copies/µl to check the analytical sensitivity and efficiency of the assays. Five plasmid dilutions (50,000–5 copies/µl) were used for evaluation of samples, which were aliquoted and stored frozen and were run in triplicate in each PCR run.

### qPCR conditions

Each 15 µl qPCR reaction mixture contained 1 × TaqMan™ Fast Advanced Master Mix (Thermo Fisher Scientific Inc.), 0.1 mg/ml bovine serum albumin (BSA) (Sigma–Aldrich), 250 nM of each primer, 100 nM of each TaqMan probe, 1 × TaqMan™ Exogenous Internal Positive Control (VIC-TAMRA labelled) Reagents (Thermo Fisher Scientific Inc.) and 2 µl template DNA. A negative control was included in each run, which consisted of nuclease-free water (W4502; Sigma–Aldrich). The qPCR program consisted of 2 min at 50 °C, 3 min at 95 °C, followed by 45 cycles of 95 °C for 3 s and 60 °C for 30 s. Amplification was performed in a CFX Opus 96 Real-Time PCR Instrument (Bio-Rad Laboratories Inc., Hercules, CA, USA) and analysed by the CFX Maestro Software version 2.0 (Bio-Rad Laboratories Inc.) with default settings. Samples for which a copy number equal to or above the limit of detection (LOD) was calculated from the qPCR assays were considered as positive.

### Modifications of the Staton et al. (2021) qPCR

The qPCR assays by Staton et al. (2021) target the recombinase A (*recA*) genes of *T. phagedenis*, *T. pedis*, and *T. medium* and are run in singleplex with the three different probes all labelled with FAM. The main modification of the qPCR in the present study was that the three probes were labelled with different fluorophores to enable multiplexing. The probe sequence for *T. phagedenis* was still labelled with FAM but the probe sequences for *T. pedis* and *T. medium* were labelled with TEX615 and Cy5, respectively. The FAM probe was double-quenched with ZEN and Iowa Black FQ, the TEX615 probe was quenched with Iowa Black RQ, and the Cy5 probe was double-quenched with TAO and Iowa Black RQ. Primers and probes for this assay were ordered from IDT. The TaqMan™ Exogenous Internal Positive Control (VIC-TAMRA labelled) Reagents (Thermo Fisher Scientific Inc.) were included as controls for possible PCR inhibition and BSA (Sigma–Aldrich) at a final concentration of 0.1 mg/ml was included to reduce the possible influence of PCR inhibitors. In addition, the same master mix, primer and probe concentrations, negative control and PCR program were used as described above in the ‘qPCR conditions’ section. The *T. phagedenis* strain V1 (Pringle et al. 2008), T. *pedis* type strain DSM 18691^T^ (Leibniz Institute DSMZ-German Collection of Microorganisms and Cell Cultures GmbH, Braunschweig, Germany), and *T. medium* type strain ATCC 700293^T^ (American Type Culture Collection, Manassas, VA, USA) were used as positive controls in each PCR run. For evaluation of analytical sensitivity and efficiency, DNA from the three strains above was combined and diluted to 32,000 copies/µl each in one tube and then ten-fold serial dilutions were made in nuclease-free water (W4502; Sigma–Aldrich) containing 1 ng/µl Polyinosinic–polycytidylic acid potassium salt (P9582; Sigma–Aldrich).

### Analytical specificity

The analytical specificity of the developed qPCR assay and the modified Staton et al. (2021) assay was evaluated on 28 *Treponema* spp. strains (Table 2) and 25 other bacterial strains (Table 3). The strains were selected on the basis of (i) close relatedness to the target species *(Treponema* spp. and *Brachyspira* spp.), (ii) species associated with BDD and CODD *(D. nodosus* and *F. necrophorum)*, and (iii) pathogenic bacteria. DNA for inclusivity and exclusivity testing was prepared using the EZ1 DNA Tissue Kit (Qiagen, Hilden, Germany) according to the manufacturer’s instructions. The extraction was performed on the EZ1 Advanced XL instrument (Qiagen) utilizing the bacterial protocol and the elution volume used was 100 µl. DNA concentrations were measured using the Qubit ds DNA Broad Range Assay Kit (Invitrogen) in a Qubit® 2.0 Fluorometer (Invitrogen). DNA preparations were diluted to 2 ng/µl in nuclease-free water (W4502; Sigma–Aldrich) prior to qPCR analysis.

**Table 2:** *Treponema* reference strains (*n* = 28) used for specificity testing of the developed triplex qPCR assay and the modified Staton et al. (2021) assay

Organism	Strain	Reference	Result triplex qPCR	Result Staton qPCR
*T. phagedenis*	V1	Pringle et al. 2008	positive *T. phagedenis*	positive *T. phagedenis*
*T. phagedenis*	V2	Rosander et al. 2011	positive *T. phagedenis*	positive *T. phagedenis*
*T. phagedenis*	T 413	Pringle et al. 2008	positive *T. phagedenis*	positive *T. phagedenis*
*T. phagedenis*	T 551B	Pringle et al. 2008	positive *T. phagedenis*	positive *T. phagedenis*
*T. phagedenis*	T 603	Pringle et al. 2008	positive *T. phagedenis*	positive *T. phagedenis*
*T. phagedenis*	T 657	Pringle et al. 2008	positive *T. phagedenis*	positive *T. phagedenis*
*T. phagedenis*	T 695	Mushtaq et al. 2016	positive *T. phagedenis*	positive *T. phagedenis*
*T. phagedenis*	T 863	Mushtaq et al. 2016	positive *T. phagedenis*	positive *T. phagedenis*
*T. phagedenis*	T 1089	Mushtaq et al. 2016	positive *T. phagedenis*	positive *T. phagedenis*
*T. phagedenis*	T 1126	Rosander et al. 2011	positive *T. phagedenis*	positive *T. phagedenis*
*T. phagedenis*	T 1138	Mushtaq et al. 2016	positive *T. phagedenis*	positive *T. phagedenis*
*T. phagedenis*	T 1237	Mushtaq et al. 2016	positive *T. phagedenis*	positive *T. phagedenis*
*T. phagedenis*	T 2378	Pringle et al. 2008	positive *T. phagedenis*	positive *T. phagedenis*
*T. pedis*	DSM 18691	Evans et al. 2009	positive *T. pedis*	positive *T. pedis*
*T. pedis*	T A4	Pringle et al. 2009	positive *T. pedis*	positive *T. pedis*
*T. pedis*	T M1	Pringle et al. 2009	positive *T. pedis*	positive *T. pedis*
*T. pedis*	B 683	Pringle & Fellström 2010	positive *T. pedis*	positive *T. pedis*
*T. pedis*	isoM1111	Karlsson et al. 2013	positive *T. pedis*	positive *T. pedis*
*T. pedis*	isoE1186	Karlsson et al. 2013	positive *T. pedis*	positive *T. pedis*
*T. pedis*	isoM1189	Karlsson et al. 2013	positive *T. pedis*	positive *T. pedis*
*T. pedis*	isoM1220	Karlsson et al. 2013	positive *T. pedis*	positive *T. pedis*
*T. pedis*	isoM1224	Karlsson et al. 2013	positive *T. pedis*	positive *T. pedis*
*T. medium*	ATCC 700293	Umemoto et al. 1997	positive *T. medium/‘T. vincentii’*	positive *T. medium*
*T. denticola*	DSM 14222	Chan et al. 1993	negative	negative
*T. parvum*	isoB1119	Karlsson et al. 2013	negative	negative
*Treponema* sp.	THI1b	Nises et al. 2018	negative	negative
*Treponema* sp.	THI4a	Nises et al. 2018	negative	negative
*Treponema* sp.	THI6	Nises et al. 2018	negative	negative

**Table 3:** Bacterial strains (*n* = 25) used for exclusivity testing of the developed triplex qPCR assay and the modified Staton et al. (2021) assay

Organism	Strain	Result triplex qPCR	Result Staton qPCR
*Actinobacillus equuli* subsp. *equuli*	CCUG 2041	negative	negative
*Aeromonas hydrophila*	CCUG 30208	negative	negative
*Brachyspira hyodysenteriae*	ATCC 27164	negative	negative
*Brachyspira pilosicoli*	ATCC 51139	negative	negative
*Brachyspira intermedia*	ATCC 51140	negative	negative
*Campylobacter coli*	CCUG 45147	negative	negative
*Campylobacter jejuni*	CCUG 11284	negative	negative
*Citrobacter freundii*	78/0309	negative	negative
*Clostridium perfringens*	CCUG 43593	negative	negative
*Dichelobacter nodosus*	AN 363/05	negative	negative
*Dichelobacter nodosus*	ATCC 25549	negative	negative
*Enterococcus faecalis*	CCUG 9997	negative	negative
*Enterococcus faecium*	CCUG 35172	negative	negative
*Escherichia coli*	CCUG 17620	negative	negative
*Fusobacterium necrophorum* subsp. *funduliforme*	CCUG 42162^T^	negative	negative
*Fusobacterium necrophorum* subsp. *necrophorum*	CCUG 9994^T^	negative	negative
*Klebsiella pneumoniae*	CCUG 45421	negative	negative
*Pasteurella multocida*	155/1909	negative	negative
*Pseudomonas aeruginosa*	CCUG 17619	negative	negative
*Rhodococcus equi*	157/1909	negative	negative
*Staphylococcus aureus*	CCUG 15915	negative	negative
*Staphylococcus intermedius*	CCUG 27191	negative	negative
*Streptococcus equi* subsp. *zooepidemicus*	CCUG 23256	negative	negative
*Streptococcus suis*	CCUG 7984^T^	negative	negative
*Streptococcus uberis*	158/1909	negative	negative

In addition, the qPCR assay developed in this study was also evaluated for specificity *in silico* on 168 *Treponema* genome assemblies, available at the National Center for Biotechnology Information (NCBI) (https://www.ncbi.nlm.nih.gov/, accessed on 15 February 2022), that had a specified species and where the 16S rDNA gene could be extracted (Suppl. Table 1). The 168 genomes represented at least 24 *Treponema* species. The three target genes: *vspA*, TPE 0673, and 16S rRNA, were extracted using the Ridom SeqSphere^+^ software version 8.3.0 (Ridom GmbH, Münster, Germany) and the respective sequences of *T. phagedenis* V2, *T. pedis* T A4 and *T. medium* ATCC 700293 were used as templates. Extracted sequences were aligned using MEGA-X version 10.2.4 (Kumar et al. 2018) and any differences to the primer and probe sequences were identified.

### Bovine digital dermatitis lesion assessment and biopsy sampling

Biopsy specimens were collected from mature dairy heifers and adult dairy cows at one abattoir located in the mid-east region of Sweden between April 12 and May 11, 2021, and November 30, 2021 and February 24, 2022. The aim was to collect biopsy specimens covering all stages of BDD, as well as specimens from cows with no signs of previous or active lesions. The interdigital skin was assessed after the lower limbs had been detached from the carcass (within 5–10 min after bleeding), and lesions were categorised using a 6-category classification scale developed by Döpfer et al. (1997) and Berry et al. (2012), with M0 = normal skin, M1 = subclinical active stage (diameter < 20 mm), M2 = clinical active stage (diameter ≥ 20 mm), M3 = healing stage, M4 = chronic stage, and M4.1 = recurrent active stage. Animals selected for M0 samples did not have DD lesions in any hoof. As the Swedish regulations do not allow for commercial transportation of lame animals, M0 and M4 were expected to be the most common lesion categories among assessed animals. To obtain a similar number of samples for each lesion category, the number of samples was restricted to a maximum of two samples per week for M0 and one sample per day for M4. The intention was to sample about 50 dairy cows but due to difficulties with admittance to abattoirs during the Covid-19 pandemic this number of samples was not achieved and there were some modifications to the original strategy (i.e., typical lesions from dairy cattle, one foot per animal): beef cattle or beef crosses were included in sampling, sampling from the same animal but different feet was performed, and some atypical lesions were included in sampling (Table 4).

**Table 4:** Biopsies collected from cattle hooves at the abattoir (LH = left hind, RH = right hind, and LF = left front) and qPCR results by sample

Sample	Lesion category	Hoof	Comment	Triplex qPCR *T. phagedenis* copy number^1^	Staton qPCR *T. phagedenis* copy number^1^
1	M0	LF		0	0
2	M0	RH		0	0
3	M0	RH		0	0
4	M0	LH	Beef cross	0	0
5	M0	LH	Beef cross	0	0
6	M0	RH		0	0
7	M0	RH		1.41E + 04	4.70E + 03
8	M0	LF		0	0
9	M1	LH		0	0
10	M1	LF		0	0
11	M1	LH		1.67E + 06	4.87E + 05
12	M1	RH	Atypical lesion	0	0
13	M2	RH		8.33E + 05	3.18E + 05
14	M2	LH		1.48E + 06	3.42E + 05
15	M2	LH	Atypical lesion	0	0
16	M3	RH		0	0
17	M4	LH		0	0
18	M4	LH		3.87E + 04	1.63E + 04
19	M4	LH		1.45E + 04	2.24E + 03
20	M4	LH		0	0
21	M4	RH	Same animal as 20	0	0
22	M4	LF		7.66E + 04	7.88E + 03
23	M4	LH	Same animal as 22	0	0
24	M4	LH		4.56E + 02	1.64E + 01
25	M4	LH		1.49E + 04	3.55E + 03
26	M4	LH		5.54E + 04	6.20E + 03
27	M4	RH	Atypical lesion	1.20E + 01	0
28	M4	LH	Atypical lesion. Same animal as 27	7.93E + 03	2.35E + 03
29	M4	LH	Atypical lesion	0	0
30	M4	RH	Atypical lesion. Same animal as 29	0	0
31	M4.1	RH		0	0
32	M4.1	LH		3.27E + 05	1.54E + 05
33	M4.1	RH		2.42E + 05	5.06E + 04
34	M4.1	RH		0	0
35	M4.1	RH		0	0
36	M4.1	RH		0	0
37	M4.1	RH		5.53E + 05	5.93E + 04

^1^Copy number per qPCR reaction (2 µl). All samples were negative for *T. pedis* and *T. medium/‘T. vincentii’*

Hooves selected for sampling were stored individually until biopsy samples could be collected (< 2 h). At sampling, organic debris in the interdigital cleft was initially wiped off with soft paper tissues and the interdigital skin was thoroughly cleaned with purified water (Millipore Milli-Q® Plus Water Purifier 18.2MΩ, Merck KGaA, Darmstadt, Germany) using one small, soft brush per hoof. The biopsy was taken fully within the lesion with a 6 mm skin biopsy punch needle (kai Europe GmbH., Solingen, Germany); 4 mm skin biopsy punch needles were used for smaller lesions. The biopsies were kept in a 1.5 or 2 ml microcentrifuge tube at room temperature and transported to the laboratory on the day of sampling where they were immediately used for DNA extraction. Prepared DNA was stored at -20 °C prior to qPCR analysis.

All assessments and biopsy collections were performed by the same trained observer (H.K.E) to ensure consistency. Before the study commenced, 60 photographs of hooves scored for DD lesions were obtained from published literature and DD scales available online (Döpfer 2014; Kofler J. 2019; Zinicola et al. 2015) to be used as a gold standard for the DD assessment training and observer evaluation. Training materials were created by randomly ordering the 60 photographs, with 14 photographs recurring three times to evaluate within-training consistency. Randomisation was obtained by numbering the photographs 1–88 and ordering them according to an integer string obtained from an online random number generator (https://www.random.org). Before data collection began, training was performed on three occasions with at least one week between sessions. For the second collection period, training was performed once before visiting the slaughterhouse. During training, the photographs were presented in the same order on each session.

The same photographs (including the repeated photographs) were also used to evaluate observer reliability during the study. Reliability was assessed using unweighted kappa (Cohen 1960) before, mid-way through and after each sample collection period. During this step, the order of the photographs was randomly changed before each assessment using the strategy described above. Observer reliability in relation to the gold standard remained high throughout the study, with kappa values ranging between 0.91 and 0.93 for the first sampling period and between 0.87 and 0.91 for the second period. Within-assessment consistency was excellent at each evaluation, showing complete agreement both within the observer and with the gold standard on all occasions.

### DNA extraction and qPCR analysis of biopsies

Bacterial DNA was extracted from the collected BDD biopsy samples using the EZ1 DNA Tissue Kit and the EZ1 DNA Bacteria Card in combination with the EZ1 Advanced XL instrument (Qiagen, Hilden, Germany) according to the manufacturer’s instructions for purification of bacterial DNA from primary samples. All biopsies were cut into small pieces (approximately 1–2 mm^3^) and pre-treated by submersion in 190 µl G2 Buffer prior to the addition of 10 µl proteinase K (600 mAU/ml) and incubation at 56 °C and 400 rpm overnight. The samples were mixed by vigorous vortexing before the addition of 1 mg Lysozyme (Merck KGaA), 50 µg Lysostaphine (Merck KGaA), and 50 units Mutanolysin (Merck KGaA), followed by incubation at 37 °C and 400 rpm for 30 min. Samples were centrifuged at 300 × g for 30 s to pellet any debris and 200 µl supernatant was transferred to a 2.0 ml sample tube and loaded into the EZ1 Advanced XL instrument (Qiagen). The elution volume used was 100 µl. Two µl of the extracted DNA was used for qPCR analysis by the developed triplex assay and the modified Staton et al. (2021) assay according to the protocols described above. The results from the two different qPCR assays were compared and the agreement between results was quantified using kappa statistics (Landis and Koch 1977). The results from each qPCR assay were also compared to lesion assessment where M0 = normal skin and M1-M4.1 = BDD lesions using the same statistics.

## Results

### Analytical sensitivity and qPCR efficiency

Plasmid dilutions of 100,000 copies to 10 copies per PCR reaction were positive for all three targets and replicates and were used to construct the standard curves to determine the LOD and amplification efficiencies for the developed qPCR assay. The LOD for the triplex qPCR assay was 10 plasmid copies per PCR reaction for all three targets. The amplification efficiencies of the *T. phagedenis, T. pedis*, and *T. medium/‘T. vincentii’* assays were 96.5% (R^2^ 0.999), 99.5% (R^2^ 0.999), and 102.6% (R^2^ 0.997), respectively.

DNA dilutions corresponding to 32,000 to 32 genome copies per PCR reaction were positive in all replicates for the *T. pedis* target in the modified Staton et al. assay (2021); hence, the LOD was 32 genome copies per PCR reaction. For *T. phagedenis* and *T. medium*, DNA dilution corresponding to 3.2 genome copies per PCR reaction was additionally positive, and for all three replicates, which gives a LOD of 3.2 genome copies per PCR reaction. The amplification efficiencies of the modified Staton et al. assay (2021), when run in multiplex were 88.1% (R^2^ 0.997), 96.5% (R^2^ 0.999), and 98.4% (R^2^ 0.997), for *T. pedis, T. phagedenis*, and *T. medium*, respectively.

### Analytical specificity of tested strains and *in silico* evaluation

The developed triplex qPCR assay showed 100% inclusivity for the 23 *T. phagedenis, T. pedis*, and *T. medium* target strains tested (Table 2) and 100% exclusivity for the 30 non-target bacterial strains (Tables 2 and 3).

In the *in silico* evaluation on 168 *Treponema* spp. genome assemblies, the *vspA* gene was detected in 12 of 15 (80%) genomes listed as *T. phagedenis* and with a 100% match of the primers and probe sequences of the *vspA* assay. The three *T. phagedenis* genome assemblies, where the *vspA* gene sequence could not be detected (AEFH01, NZ_CP031394.1 and VOQA01), were all of human origin. The *vspA* gene was not detected in any of the 153 non-*T. phagedenis* genomes. The TPE 0673 gene was found in 9 of 9 (100%) *T. pedis* genomes and in none of the 159 genomes from *Treponema* species other than *T. pedis.* There was a 100% match of the primers and probe sequences of the TPE 0673 assay and the *T. pedis* genomes, except for AOTP01, where there was one mismatch in the middle of the reverse primer. The 16S rRNA primers and probe were 100% conserved for the three *T. medium* genomes and for three of four *‘T. vincentii’* genomes investigated. One of the *‘T. vincentii’* genomes (ACYH01) had a 2-bp mismatch in the forward primer. In addition, there was a 100% match of the primer and probe sequences for *Treponema* OMZ 838 (NZ_CP009227), which, according to Chan et al. (2014), is *T. medium/‘T. vincentii’*. For the remaining 160 *Treponema* genomes, the primer and probe sequences were conserved between 53.5% and 81.7% (Suppl. Table 2). Of the non-target *Treponema*-species, *T. denticola* had the highest mean sequence conservation in the primer and probe regions (81.7%), compared with 99.6% on average for the target species (Suppl. Table 2).

### qPCR evaluation of biopsies

**Fig. 1:**
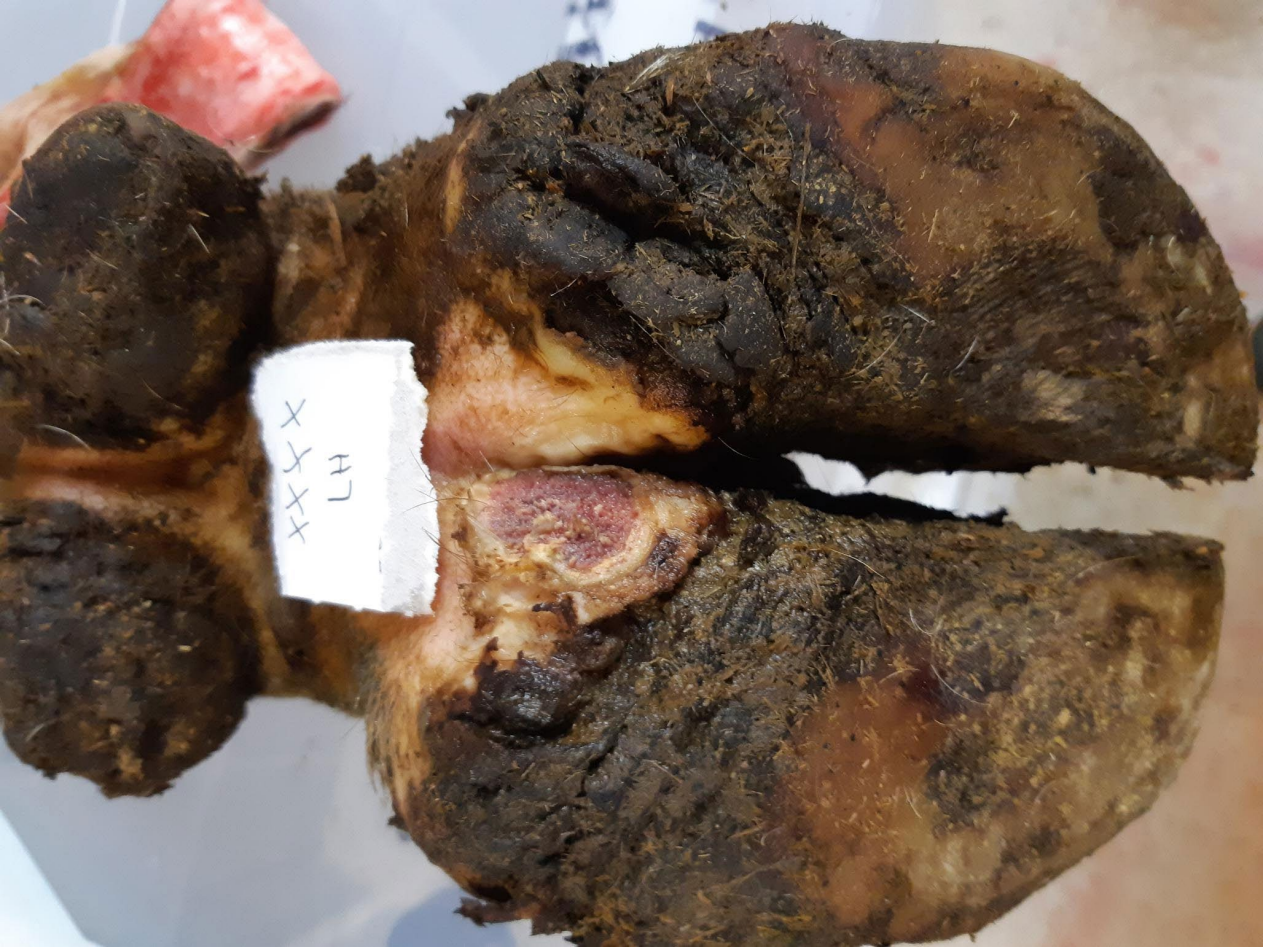
Typical appearance of cattle hoof with M2 lesion found in this study

In total, 37 cattle hooves were sampled at the abattoir and the distribution between different lesion categories was as follows: M0 = 8, M1 = 4, M2 = 3 (Fig. 1), M3 = 1, M4 = 14, and M4.1 = 7 (Table 4). At least one sample from each lesion category was positive for *T. phagedenis* in the developed triplex qPCR assay and in the modified Staton et al. (2021) assay except for lesion category M3, which only contained one sample (Table 5). *Treponema phagedenis* qPCR copy number results at sample level are presented in Table 4. *Treponema pedis* and *T. medium/‘T. vincentii’* were not detected in any lesion category. A difference between the two qPCR assays was noted for lesion category M4, where 8 out of 14 samples were positive in the developed triplex qPCR assay compared to 7 samples in the Staton et al. (2021) qPCR assay. A kappa value of 0.94 (95% CI 0.83-1.00) indicated almost perfect agreement between the two qPCR assays. Kappa values of 0.22 (95% CI 0.00 to 0.44) and 0.19 (95% CI -0.01 to 0.40) were obtained for the developed triplex qPCR and the modified Staton et al. (2021) assays, respectively, when compared to lesion assessment. This indicated fair agreement and slight agreement, respectively.

**Table 5:** Evaluation of the developed triplex qPCR assay and the modified Staton et al. (2021) assay on DNA from bovine digital dermatitis biopsy samples, by lesion category

Lesion category	Number of samples	Result triplex qPCR^1^	Result Staton qPCR^1^
M0	8	1 positive *T. phagedenis*	1 positive *T. phagedenis*
M1	4	1 positive *T. phagedenis*	1 positive *T. phagedenis*
M2	3	2 positive *T. phagedenis*	2 positive *T. phagedenis*
M3	1	0 positive *T. phagedenis*	0 positive *T. phagedenis*
M4	14	8 positive *T. phagedenis*	7 positive *T. phagedenis*
M4.1	7	3 positive *T. phagedenis*	3 positive *T. phagedenis*
**Total**	**37**	**15 positive ** ***T. phagedenis***	**14 positive ** ***T. phagedenis***

^1^ Positive signals in both the developed triplex qPCR assay and the modified Staton et al. (2021) assay arose from the same DNA samples, except for the one sample originating from a lesion category M4 biopsy that only gave a signal in the developed triplex qPCR assay and not the modified Staton et al. (2021) assay

## Discussion

In this study, a multiplex qPCR assay targeting *T. phagedenis*, *T. pedis*, and *T. medium/‘T. vincentii’* was developed and evaluated on BDD biopsies. An early and accurate diagnosis of BDD is important for correct and effective measures to be implemented to reduce transmission of BDD. Given the similar bacteriological profile of BDD and CODD, especially with regard to *Treponema* spp. (Duncan et al. 2021; Staton et al. 2021), such an assay could most likely also be used on CODD samples. At the start of this study, there was no qPCR method that met our requirements for this purpose. When another qPCR method was published during the course of the study (Staton et al. 2021), we chose to modify it so that it could be run in multiplex and compared with ours. The modification had no major impact on the analytical sensitivity of the assay, and thus it is not likely that different results would have been obtained for the clinical samples if the method had been run as singleplex.

The *vspA* gene used as a target for the detection of *T. phagedenis* in the triplex qPCR developed in this study was shown to be present in 12 out of 15 *T. phagedenis* genomes in the *in silico* evaluation. This is consistent with our previous results that the gene is not present in the human-originating *T. phagedenis* genomes available today (Mushtaq et al. 2016). The *vspA* gene was shown to be part of a locus with genes encoding three potential phase variable antigenic proteins, and as such they may play a role in the pathogenesis of *T. phagedenis*. Therefore, the qPCR developed in this study may be more selective than other PCR assays developed for detection of *T. phagedenis* with regard to origin and pathogenesis.

Both qPCR assays showed 100% specificity when tested on bacterial strains. The analytical sensitivity was good and comparable between the two methods. The agreement between the two methods was very good (kappa = 0.94) when tested on BDD biopsies. However, agreement with lesion assessment was poor. The qPCR developed in this study detected 3.4% more *T. phagedenis*-positive biopsies of lesion category M1-M4.1 than the modified Staton et al. (2021) assay. However, none of the methods gave positive results for more than 51.7% of the M1-M4.1 biopsies, and only *T. phagedenis* was detected. The samples for which *T. phagedenis* was detected were the same in both assays except for one lesion category M4 biopsy, where *T. phagedenis* was detected with the qPCR developed in this study but not in the modified Staton et al. (2021) assay. *Treponema phagedenis* is the only treponemal species that has been cultured from cases of BDD in Sweden (Mushtaq et al. 2016), whereas in other countries *T. pedis* and *T. medium* are frequently isolated alongside with *T. phagedenis* (Brodard et al. 2021; Evans et al. 2009). However, it cannot be ruled out that other *Treponema* species could be present in these BDD biopsies. At least 20 different phylotypes of the genus *Treponema* have been found in BDD lesions (Klitgaard et al. 2013). Metagenomic studies have revealed the presence of different treponemes in different lesion stages where *T. phagedenis* is most common (Krull et al. 2014; Nielsen et al. 2016). As of yet, there is no deep sequencing-based data, or similar, available on the treponemal or bacterial composition of the microbiota in Swedish BDD lesions. Another possible explanation for the low detection rate could be due to the fact that some of the sampled lesions were atypical. The few sampled lesions from beef cross cattle (*n* = 2) most likely do not lead to bias as beef BDD lesion microbiota have been shown not to have drastic differences compared to the dairy BDD lesion microbiota (Caddey et al. 2021). Also, a lower association of *T. pedis* and *T. medium/‘T. vincentii’* with Swedish BDD lesions could be the result of tissue DNA extraction and handling. Future studies including more clinical samples should help resolve what is the cause of the lower association. Both assays detected *T. phagedenis* in 12.5% of the M0 biopsies, which is consistent with another study (Beninger et al. 2018). It is possible that cattle with M0 and positive results for *T. phagedenis* could eventually develop BDD. A longitudinal study would be needed to investigate this, which was not possible in this study since we used hooves from an abattoir.

To conclude, the triplex qPCR assay developed in the present study to detect *T. phagedenis*, *T. pedis*, and *T. medium/‘T. vincentii’* has high analytical sensitivity and specificity and provides a useful complementary tool for diagnosis and epidemiological studies of BDD and possibly CODD. The benefits of the robust triplex qPCR are cost-saving, with fewer reactions required, as well as time-saving, allowing an enhanced throughput of samples. The developed qPCR is, however, not intended as a replacement for clinical diagnosis as the aetiology of BDD and CODD has not been completely established and a full evaluation of the method with more clinical samples has yet to be performed.

### Electronic supplementary material

Below is the link to the electronic supplementary material.

Supplementary Material 1

Supplementary Material 2

## Data Availability

The datasets used and/or analysed during the current study are available from the corresponding author on reasonable request.
